# Supplementary material for: iNOS is necessary for GBP-mediated T. gondii clearance in murine macrophages via vacuole nitration and intravacuolar network collapse
Source: Nat Commun. 2024 Mar 27;15:2698. doi: 10.1038/s41467-024-46790-y (PMC10973475; doi:10.1038/s41467-024-46790-y)
Supplement: Supplementary file 1 — Supplementary Information [file 41467_2024_46790_MOESM1_ESM.pdf]

iNOS is necessary for GBP-mediated *T. gondii* clearance in murine macrophages via vacuole nitration and intravacuolar network collapse

Xiao-Yu Zhao[1], Samantha L. Lempke[1], Jan C. Urbán Arroyo[1], Isabel G. Brown[1], Bocheng Yin[1], Magdalena M. Magaj[2], Nadia K. Holness[1], Jamison Smiley[1], Stefanie Redemann[2], Sarah E. Ewald[1*]

Affiliations

[1]Department of Microbiology, Immunology, and Cancer Biology at the Carter Immunology Center, University of Virginia School of Medicine, Charlottesville, Virginia, USA

[2]Center for Membrane and Cell Physiology, Department of Molecular Physiology and Biological Physics, University of Virginia School of Medicine, Charlottesville, Virginia, USA

[*]Correspondence: se2s@virginia.edu (S.E.E)

Supplementary Fig. 1

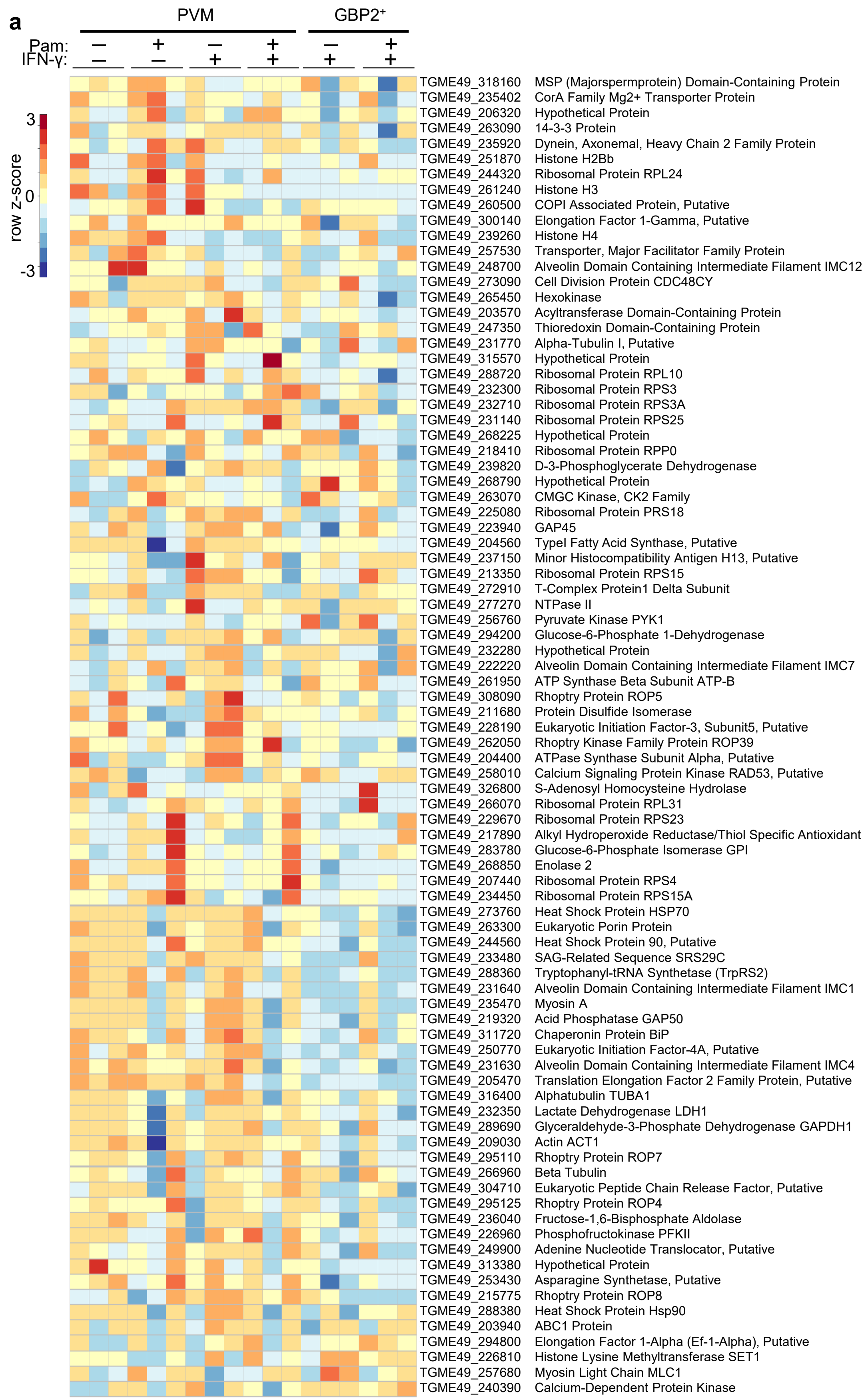

Supplementary Fig. 1 (related to Fig.2). *T. gondii* proteins identified by autoSTOMP.
a. 86 *T. gondii* proteins were identified using autoSTOMP as described in Fig. 2

Supplementary Fig. 2

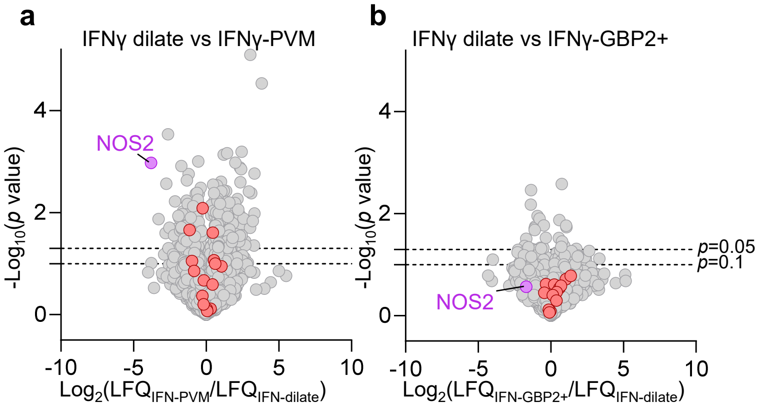

Supplementary Fig. 2 (related to Fig.2).

Pair-wise comparison of dilate and PVM samples from IFNγ treated BM-mDCs.

Pair-wise comparison of IFNγ dilate versus IFNγ PVM (**a**) or IFNγ dilate versus IFNγ GBP2$^+$ (**b**). IRG and GBP proteins (red circle) and iNOS (purple circle) were labeled. IIGs, red; iNOS, violet. Plots showing $-\log_{10}$(*p* value) from Student's t test and $\log_2$LFQ differences between each comparison. Dotted lines indicated *p*=0.1 and *p*=0.05 respectively.

Supplementary Fig. 3

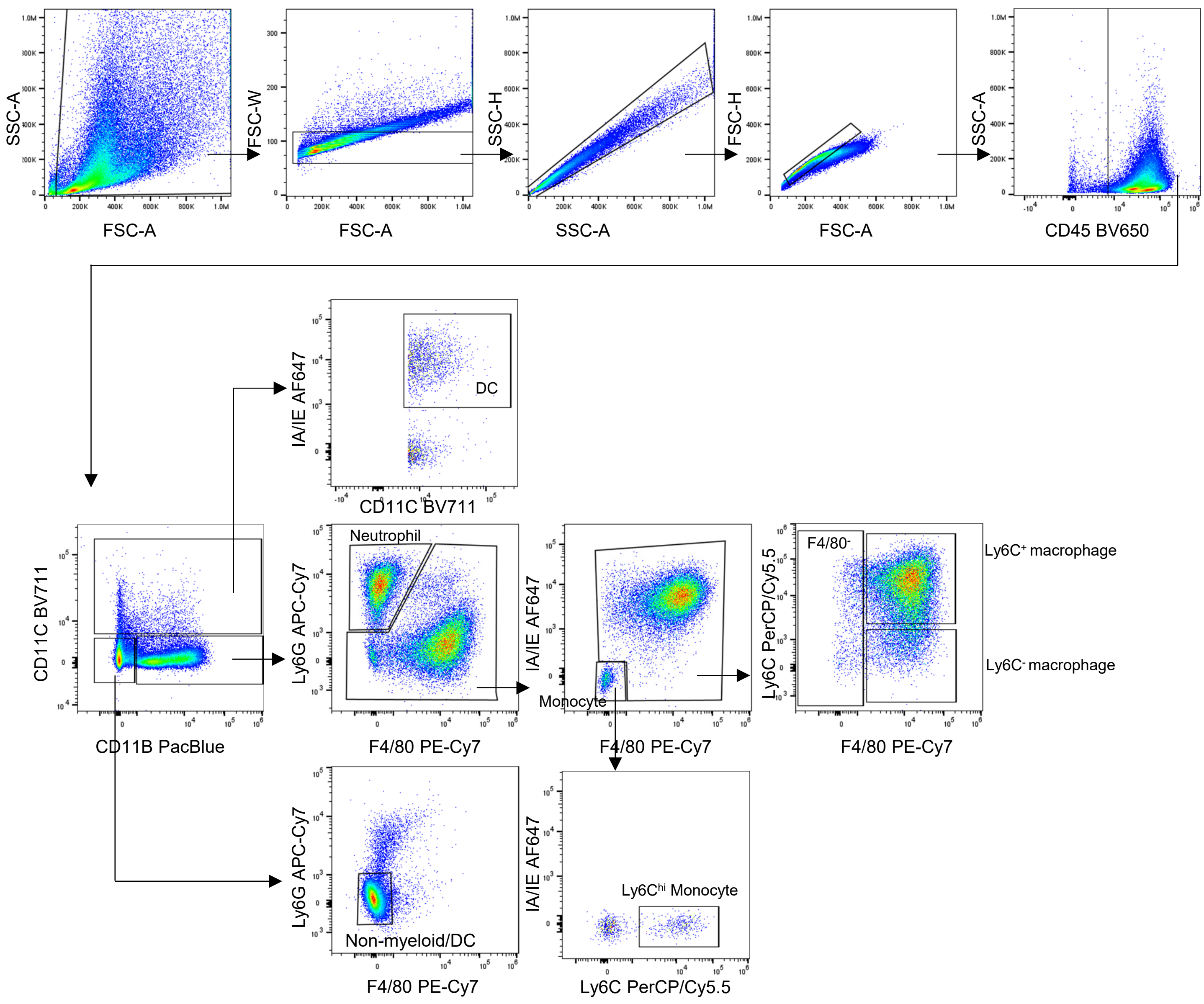

Supplementary Fig. 3 (related to Fig. 3). Peritoneal exudate cell flow cytometry gating strategy
a. Gating strategy for Fig. 3.

Supplementary Fig. 4

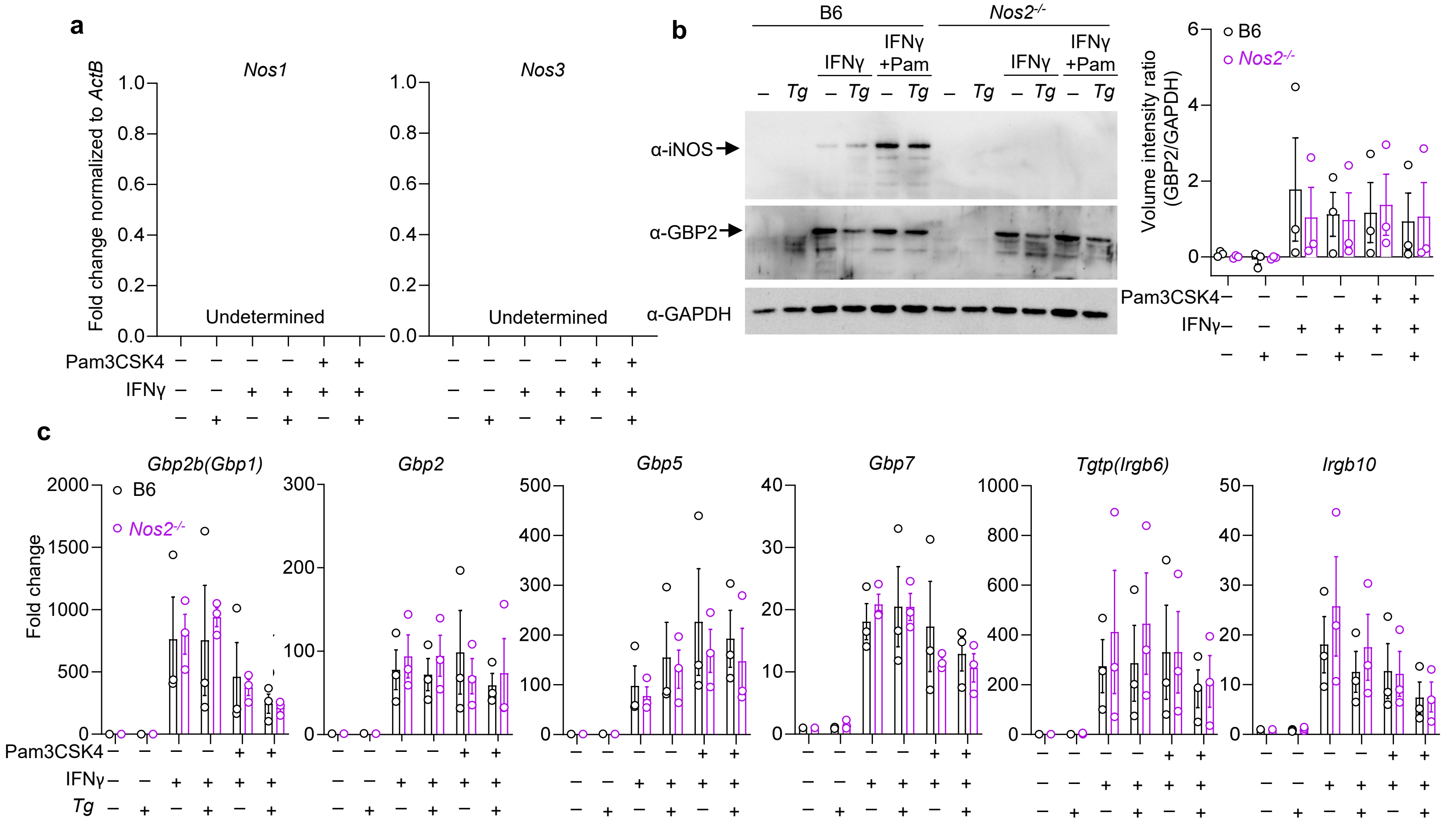

Supplementary Fig. 4 (related to Fig. 4). Expression of nitric oxide synthases and IIGs in BM-MoDCs.

a, B6 BM-MoDCs were infected as described in Fig. 1. *Nos1* and *Nos3* expression was determined using quantitative reverse transcription polymerase chain reaction (RT-PCR) at 2hpi. N=3 independent experiments. **b,** GBP2 protein levels were detected by Western blot at 14hpi respectively. Volumetric intensity ratios of GBP2 and GAPDH were quantified (right). **c**, IIG expression was determined using quantitative reverse transcription polymerase chain reaction (RT-PCR) at 2 hours post infection. N=3 independent experiments.

Supplementary Fig. 5

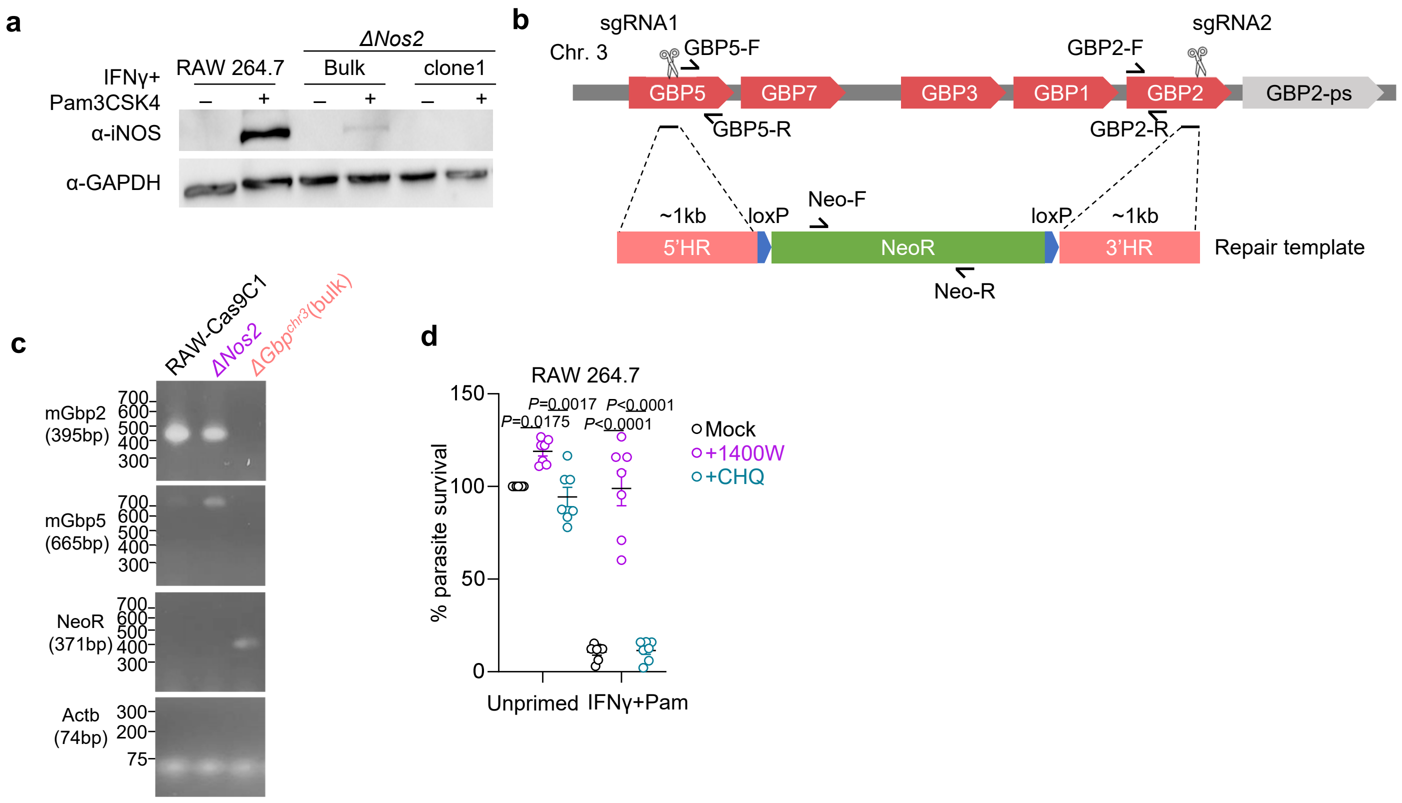

Supplementary Fig. 5 (related to Fig. 4). RAW-Cas9 deletion of iNOS and chromosome 3 GBPs.

a, iNOS expression was interrupted by CRISPR/Cas9 in Cas9-expressing RAW 264.7 macrophages and iNOS expression was evaluated in the bulk population or a single cell clone (clone 1) by western blot. **b**, Schematic of the chromosome 3 GBP locus and strategy for generating RAW$^{\Delta Gbp\text{-}chr3}$ cells using CRISPR-Cas9 mediated homologous recombination. **c,** Validation of neomycin resistance cassette integration in chromosome 3 genomic DNA, loss of GBP5 downstream of the integration site and loss of GBP2 upstream of the integration site in the bulk population of RAW$^{\Delta Gbp\text{-}chr3}$ cells (bulk population). **d,** RAW 264.7 macrophages were pretreated for 20 hours with media, 10ng/mL IFNγ and 10ng/mL of Pam3CSK4 for the final 3 hours before infection. Cells were infected with *T. gondii* expressing GFP and luciferase at MOI 5. Cells were pretreated with 1400W 1hr before infection or chloroquine (CHQ) 30 minutes post infection. N=7 independent experiments, 2-way ANOVA with Tuckey post hoc analysis.

Supplementary Fig. 6

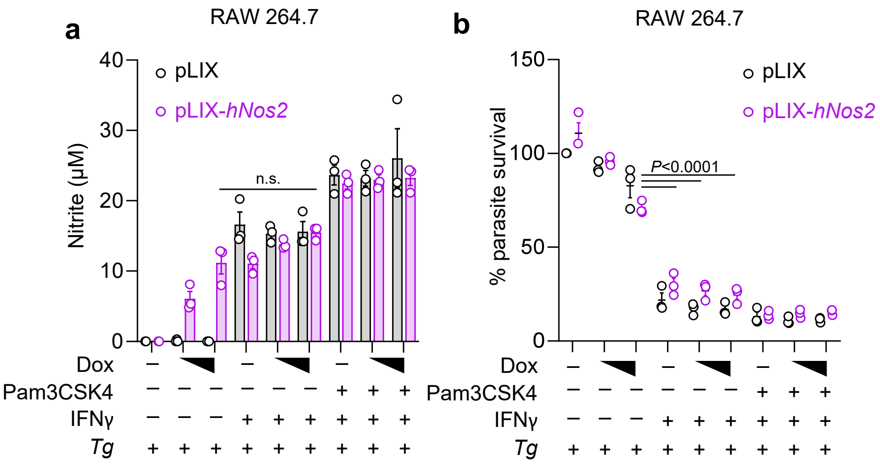

Supplementary Fig. 6 (related to Fig. 5). iNOS overexpression cannot restrict parasites in the absence of IFNγ.
a-b, RAW 264.7 macrophages were transduced with human *Nos2* under tetracycline inducible promoter and human iNOS expression was titrated by adding no, 200 ng/mL or 400 ng/mL doxycycline which was much lower that OD50 of doxycycline for *T. gondii*. Cells were infected similar to Fig. 4 and nitrite production (**a**) was measured to probe for nitric oxide flux while parasite burden was measured by luciferase assay (**b**). N=3 independent experiments, and error bars represent Mean±SEM by 2-way ANOVA with Tukey post hoc analysis. n.s., not significant.

Supplementary Fig. 7

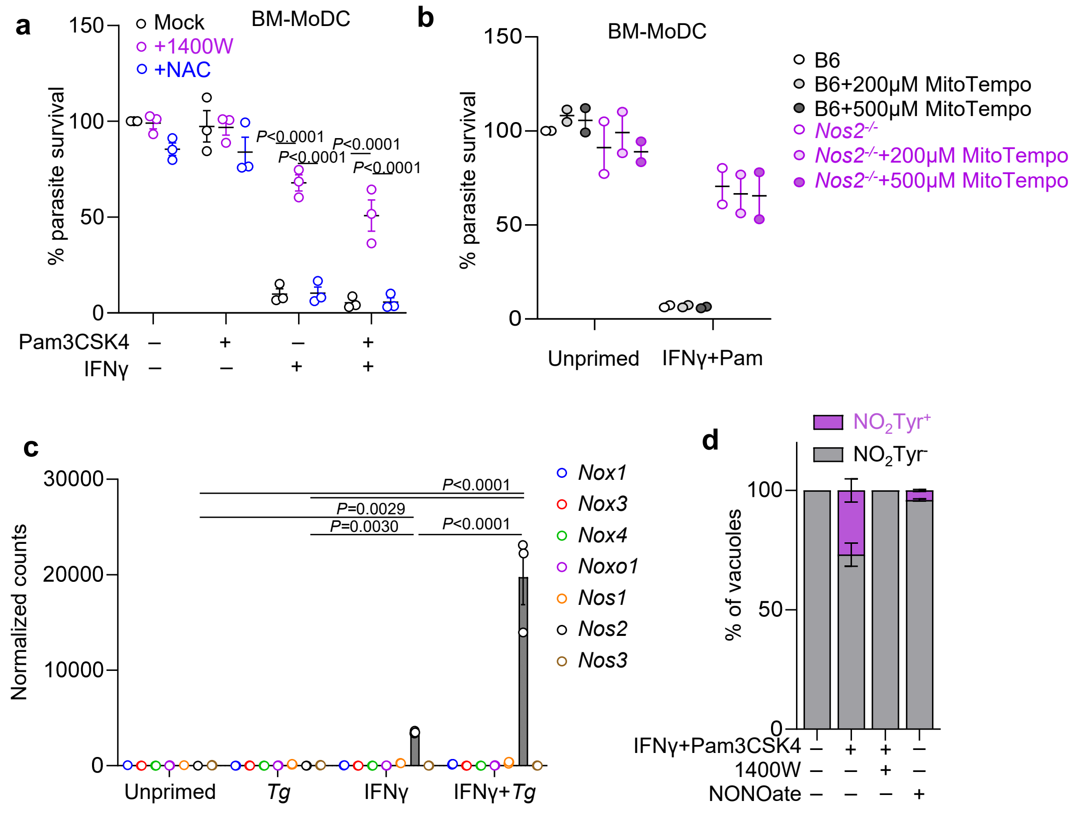

Supplementary Fig. 7 (related to Fig. 5). Inhibiting cellular or mitochondrial ROS does not rescue parasite growth downstream of IFNγ and Pam3CSK4 stimulation, and NO supplementation can not nitrate the vacuole in unstimulated macrophages

a-b, BM-MoDCs were stimulated and infected as described in Fig. 1. Parasite growth in mock treated or media supplemented with iNOS inhibitor, 1400W, cellular ROS inhibitor, N-acetyl cysteine (NAC) (**a**) or mitochondrial targeted ROS scavenger, mitoTEMPO (**b**) was measured by luciferase assay at 14 hpi. 2-way ANOVA with Tuckey post hoc analysis. **c-d**, RAW 264.7 cells were infected with Me49-GFP-Luc as described in Fig. 4. **c,** RNAseq analysis of RAW 264.7 cells at 18hpi. **d,** The NO donor DETA NONOate was added to RAW 264.7 cells one hour prior to infection, and samples were fixed and stained with a nitrotyrosine-specific (NO_2-Y, magenta) antibody to evaluate co-localization with parasite GFP. Error bars in **c** represent Mean±SEM by 2-way ANOVA with Tukey post hoc analysis.

Supplementary Fig. 8

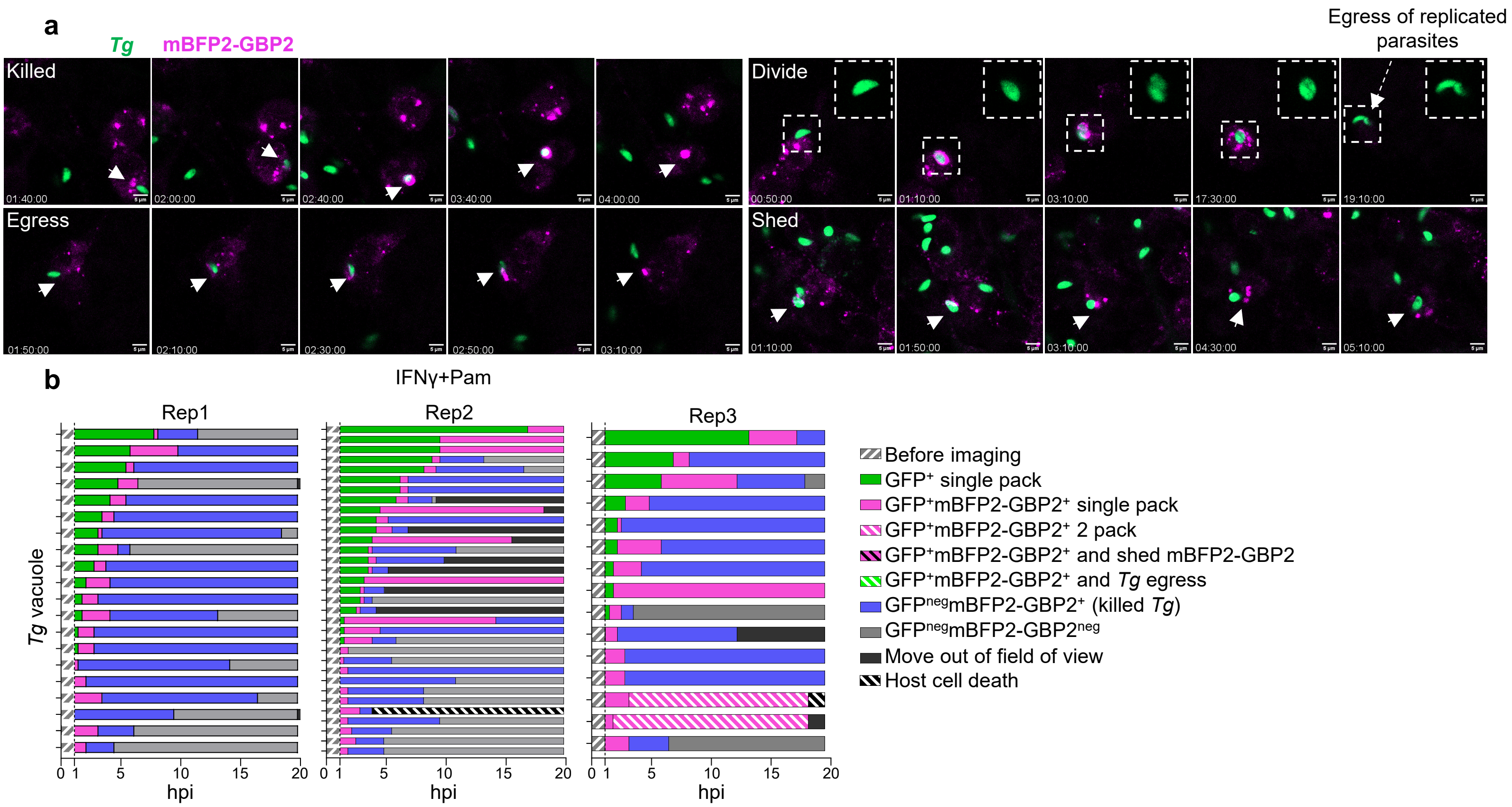

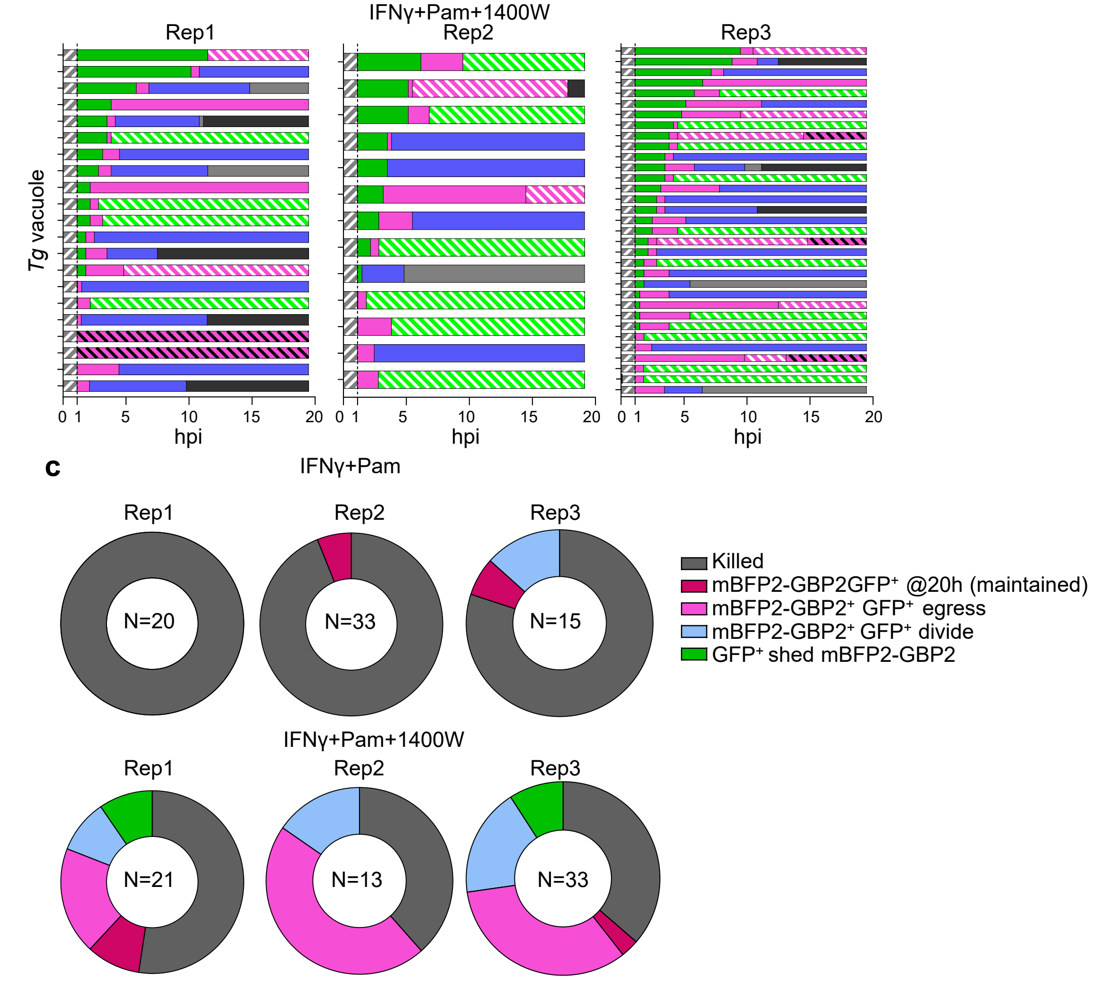

Supplementary Fig. 8 (related to Fig. 6). iNOS inhibtion facilitates replication, egress, and GBP shedding *T. gondii* vacuoles targeted by GBP2.
RAW-Cas9 macrophages expressing GBP2 fused to a blue fluorescent protein, mTag-BFP2, (mBFP2-GBP2) stimulated with IFNγ and Pam3CSK4 alone or with 1400W as described in Figure 4 then infected with Me49-GFP-luc MOI=5. Images were collected every 20 minutes for 20 hours. **a.** Representative images of GBP2-BFP targeting and Me49-GFP-luc signal. **b.** Quantification of mBFP2-GBP2 -targeted *T. gondii* vacuoles. The following phenotypes were quantified on each frame: mBFP2-GBP2⁻ GFP⁺ (green), mBFP2-GBP2⁺GFP⁺ (pink), mBFP2-GBP2 ⁺ that lost GFP signal (blue), parasites that lost GFP and mBFP2-GBP2 signal (grey), mBFP2-GBP2 ⁺ GFP⁺ parasites that divide (pink/white stripes), GFP⁺ parasites that strip mBFP2-GBP2 signal (pink/black stripe), GFP⁺ parasites that lost mBFP2-GBP2 signal via egress from host cells (green stripe). Black indicates parasites that migrate out of field of view, black hatch indicates host cell death. **c.** For data shown in (**b**) total number of parasites that lost GFP signal with morphological changes consistent with loss of viability (grey), parasites that remained GFP⁺ and mBFP2-GBP2⁺ by the end of imaging (red), GFP⁺ mBFP2-GBP2⁺ parasites that lost GBP2-BFP signal via egress (pink). GFP⁺ GBP2-BFP⁺ parasites that divided (blue), GFP⁺ and GBP2-BFP⁺ parasites that shed GBP2-BFP signal (green). Data represent 3 independent experiments, IFNγ and Pam3CSK4 N=68 vacuoles; IFNγ and Pam3CSK4 plus 1400W N=67 vacuoles.

Supplementary Fig. 9

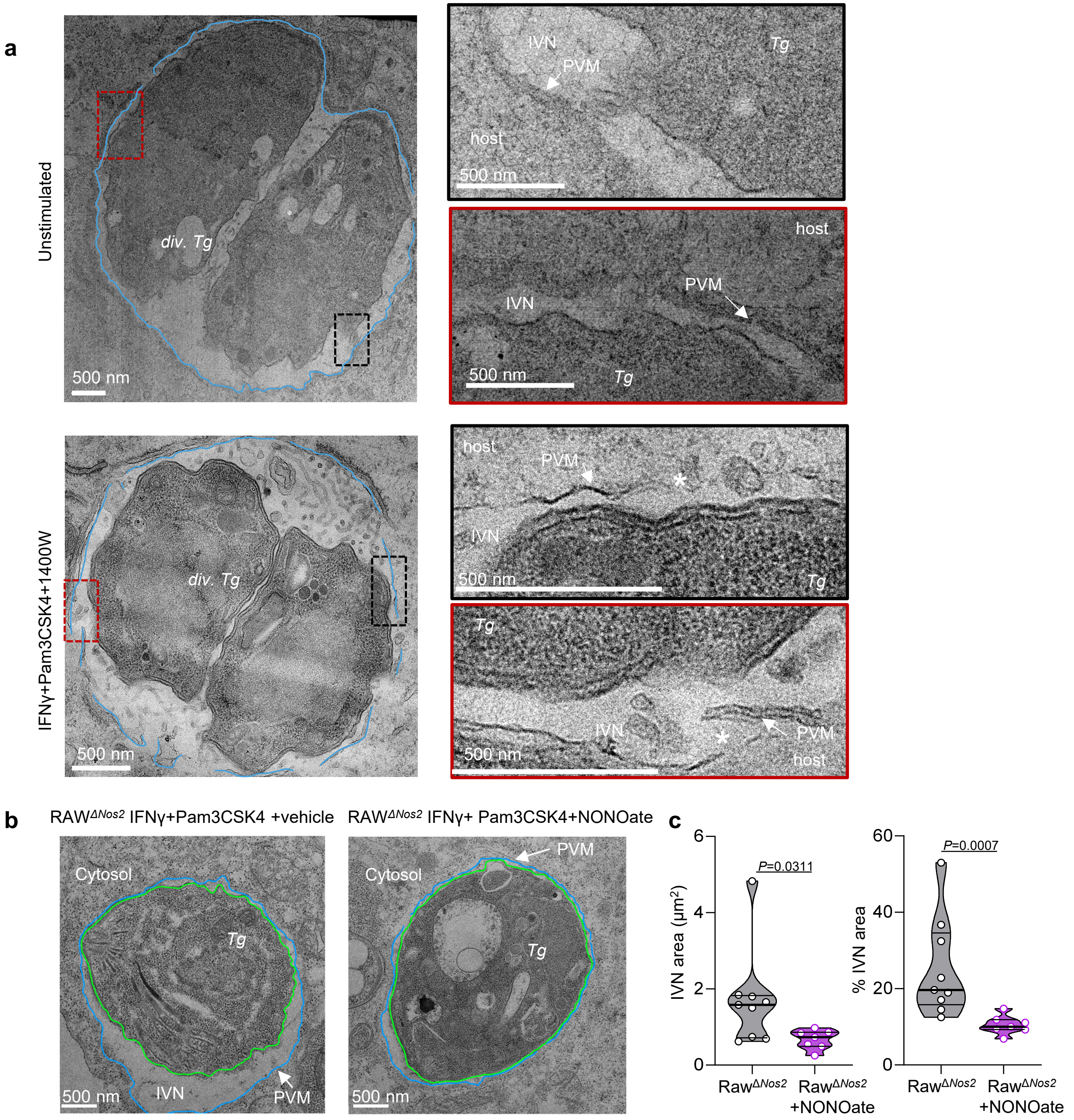

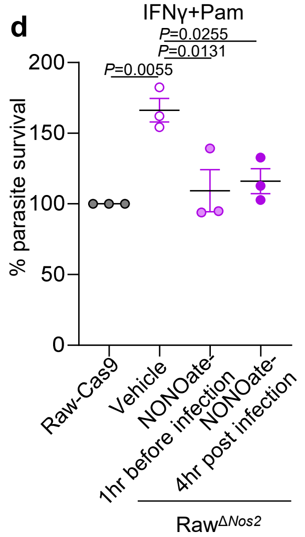

Supplementary Fig. 9 (related to Fig. 6). PVM ultrastructure of 2 pack vacuole in IFNγ and Pam3CSK4 stimulated RAW 264.7 cells during iNOS inhibition.

a, Vacuole ultrastructure was evaluated by transmission electron microscopy in RAW-Cas9 cells that were unstimulated, treated with IFNγ and Pam3CSK4 with 1400W then infected for 6 hours. Insets show the intravacuolar network (IVN) between the parasite plasma membrane (*Tg*) and the parasitophorous vacuole membrane (PVM, blue line), * indicates breaks in the PVM, *div. Tg* indicates vacuoles with 2 parasites. **b.** Representative images of vacuole ultrastructure evaluated by transmission electron microscopy. RAW$^{\Delta Nos2}$ cells were treated with IFNγ and Pam3CSK4 and infected for 6 hours then incubated with vehicle or 300μM DETA NONOate for 6 hours. **c.** Quantification of the area between the PVM and parasite plasma membrane in microns (left, IVN area) or shown as the percent of the total vacuole (IVN and parasite) volume (right) in vehicle control (n=9) and NONOate treated (n=7) cells. Mann Whitney test, violin plots showing median with quartiles. **d.** RAW or RAW$^{\Delta Nos2}$ cells were prestimulated and treated with NONOate before infection, as described in Figure 4, or NONOate was spiked in 4 hours post-infection. Parasite burden was measured by luciferase assay at 14 hpi. N=3 independent experiments. One way ANOVA with Tuckey post hoc analysis.

Supplementary Table 1: Antibodies used in this study

Antibody	Source	Catalog number	Application	Dilution	Validation
α-GBP2	Proteintech	11854-1-AP	Immunofluorescence	1:500	https://www.ptglab.com/products/GBP2-Antibody-11854-1-AP.htm
α-Toxoplasma polyclonal, FITC	Thermo Fisher Scientific	PA1-7253	Immunofluorescence	1:500	https://www.thermofisher.com/antibody/product/Toxoplasma-gondii-Antibody-Polyclonal/PA1-7253
α-TgSAG1 (D61S)	Thermo Fisher Scientific	MA518268	Immunofluorescence	1:100	https://www.thermofisher.com/antibody/product/Toxoplasma-gondii-SAG1-Antibody-clone-D61S-Monoclonal/MA5-18268
α-iNOS	BD Biosciences	610328	Immunofluorescence	1:500	https://www.bdbiosciences.com/en-us/products/reagents/microscopy-imaging-reagents/immunofluorescence-reagents/purified-mouse-anti-inos-nos-type-ii.610328
α-nitrotyrosine	EMD Millipore	06-284	Immunofluorescence	1:200	https://www.emdmillipore.com/US/en/product/Anti-Nitrotyrosine-Antibody,MM_NF-06-284
Goat anti-Rabbit IgG (H+L) Highly Cross-Adsorbed Secondary Antibody, Alexa Fluor 594	Thermo Fisher Scientific	A11037	Immunofluorescence	1:500	https://www.thermofisher.com/antibody/product/Goat-anti-Rabbit-IgG-H-L-Highly-Cross-Adsorbed-Secondary-Antibody-Polyclonal/A-11037
Donkey anti Rabbit IgG (H+L) Highly Cross Adsorbed Secondary Antibody, Alexa Fluor 647	Thermo Fisher Scientific	A31573	Immunofluorescence	1:500	https://www.thermofisher.com/antibody/product/Donkey-anti-Rabbit-IgG-H-L-Highly-Cross-Adsorbed-Secondary-Antibody-Polyclonal/A-31573
AffiniPure Donkey Anti Mouse IgG (H+L), Alexa Fluor 594	Jackson ImmunoResearch	715-585-150	Immunofluorescence	1:500	https://www.jacksonimmuno.com/catalog/products/715-585-150
CD11b-Pacific Blue	BioLegend	101224	Flow cytometry	1:200	https://www.biolegend.com/en-ie/products/pacific-blue-anti-mouse-human-cd11b-antibody-3863?GroupID=BLG10552
CD45-BV650	BioLegend	103151	Flow cytometry	1:200	https://www.biolegend.com/en-ie/products/brilliant-violet-650-anti-mouse-cd45-antibody-11987
CD11c-BV711	BioLegend	117349	Flow cytometry	1:200	https://www.biolegend.com/de-de/products/brilliant-violet-711-anti-mouse-cd11c-antibody-10175?GroupID=BLG11937
Ly6C-PerCP/Cy5.5	BioLegend	128012	Flow cytometry	1:200	https://www.biolegend.com/nl-be/products/percp-cyanine5-5-anti-mouse-ly-6c-antibody-5967?GroupID=BLG5853
F4/80-PE/Cy7	BioLegend	123113	Flow cytometry	1:200	https://www.biolegend.com/en-ie/products/pe-cyanine7-anti-mouse-f4-80-antibody-4070?GroupID=BLG5319
I-A/I-E-AF647	BioLegend	107618	Flow cytometry	1:200	https://www.biolegend.com/nl-be/products/alexa-fluor-647-anti-mouse-i-a-i-e-antibody-3135?GroupID=BLG11931
Ly6G-APC/Cy7	BioLegend	127624	Flow cytometry	1:200	https://www.biolegend.com/en-ie/products/apc-cyanine7-anti-mouse-ly-6g-antibody-6755?GroupID=BLG5803
iNOS-AF594	BioLegend	696803	Flow cytometry	1:200	https://www.biolegend.com/fr-fr/products/alexa-fluor-594-anti-nos2-antibody-15477
α-GBP2	Proteintech	11854-1-AP	Western blot	1:1000	https://www.ptglab.com/products/GBP2-Antibody-11854-1-AP.htm
α-iNOS	BD Biosciences	610328	Western blot	1:1000	https://www.bdbiosciences.com/en-us/products/reagents/microscopy-imaging-reagents/immunofluorescence-reagents/purified-mouse-anti-inos-nos-type-ii.610328
α-GAPDH	Cell Signaling Technology	5174	Western blot	1:1000	https://www.cellsignal.com/products/primary-antibodies/gapdh-d16h11-xp-rabbit-mab/5174
Peroxidase AffiniPure Goat Anti-Mouse IgG	Jackson ImmunoResearch	115-035-003	Western blot	1:10,000	https://www.jacksonimmuno.com/catalog/products/115-035-003
Peroxidase AffiniPure Donkey Anti-Rabbit IgG	Jackson ImmunoResearch	711-035-152	Western blot	1:10,000	https://www.jacksonimmuno.com/catalog/products/711-035-152
