# Supplementary material for: iNOS is necessary for GBP-mediated T. gondii clearance in murine macrophages via vacuole nitration and intravacuolar network collapse
Source: Nat Commun. 2024 Mar 27;15:2698. doi: 10.1038/s41467-024-46790-y (PMC10973475; doi:10.1038/s41467-024-46790-y)
Supplement: Supplementary file 4 — Description of Additional Supplementary Files [file 41467_2024_46790_MOESM4_ESM.docx]

**Description of additional supplementary files**

Supplementary Data 1: **All identified mouse proteins in autoSTOMP and pairwise comparisons between autoSTOMP conditions**

Log_2_LFQ values from autoSTOMP conditions and -log_10_*p* values from two-sided Student *t* test was provided. Data for pairwise comparisons presented in the paper were included in the separate tabs in this file.

Supplementary Data 2: **All identified *T. gondii* proteins in autoSTOMP**

Log_2_LFQ values of *T. gondii* proteins from autoSTOMP conditions

Supplementary Data 3: **Primers used in this study and plasmid sequence**

Supplementary Movie 1: **A killed parasite targeted by mBFP2-GBP2**

Time-lapse of RAW-Cas9 cells expressing inducible GBP2 fused to mTagBFP2 (mBFP2-GBP2) primed with IFNγ and Pam3CSK4 alone or with 1400W then infected with Me49-GFP-luc. GFP^+^ parasites targeted by mBFP2-GBP2 were quantified. Images were taken every 20 minutes between 1 and 20 hpi. This movie shows a parasite targeted by mBFP2-GBP2 and eventually killed.

Supplementary Movie 2: **A mBFP2-GBP2 targeted parasite that egressed**

Time-lapse of RAW-Cas9 cells expressing inducible GBP2 fused to mTagBFP2 (mBFP2-GBP2) primed with IFNγ and Pam3CSK4 alone or with 1400W then infected with Me49-GFP-luc. GFP^+^ parasites targeted by mBFP2-GBP2 were quantified. Images were taken every 20 minutes between 1 and 20 hpi. This movie shows a parasite targeted by mBFP2-GBP2 and egressed.

Supplementary Movie 3: **A mBFP2-GBP2 targeted parasite that divided**

Time-lapse of RAW-Cas9 cells expressing inducible GBP2 fused to mTagBFP2 (mBFP2-GBP2) primed with IFNγ and Pam3CSK4 alone or with 1400W then infected with Me49-GFP-luc. GFP^+^ parasites targeted by mBFP2-GBP2 were quantified. Images were taken every 20 minutes between 1 and 20 hpi. This movie shows a parasite targeted by mBFP2-GBP2 that divided and then egressed.

Supplementary Movie 4: **A mBFP2-GBP2 targeted parasite that shed mBFP2-GBP2 and then divided**

Time-lapse of RAW-Cas9 cells expressing inducible GBP2 fused to mTagBFP2 (mBFP2-GBP2) primed with IFNγ and Pam3CSK4 alone or with 1400W then infected with Me49-GFP-luc. GFP^+^ parasites targeted by mBFP2-GBP2 were quantified. Images were taken every 20 minutes between 1 and 20 hpi. This movie shows a parasite targeted by mBFP2-GBP2 that shed mBFP2-GBP2 and divided.
